# Psychometric refinement of the study-related perfectionism scale in Chinese adolescents: fairness and longitudinal evidence for a four-item concerns-focused adaptation (SPS-4-CV)

**DOI:** 10.3389/fpsyg.2026.1789348

**Published:** 2026-07-03

**Authors:** Weizhe Chen, Zhihui Fu, Xuhuihong Cheng, Jiani Cai, Cheng Min, Zhiwen Zhao

**Affiliations:** 1 School of Education and Psychology, Minnan Normal University, Zhangzhou, Fujian, China; 2 Fujian Key Laboratory of Granular Computing and Applications, Zhangzhou, Fujian, China; 3 School of Mathematics and Statistics, Minnan Normal University, Zhangzhou, Fujian, China; 4 Nanlin Middle School, Tongshan County, Xianning, Hubei, China; 5 School of Psychology, Inner Mongolia Normal University, Hohhot, Inner Mongolia, China; 6 School of Education, Hebei Normal University, Shijiazhuang, Hebei, China; 7 School of Mathematics and Statistics, Changchun University of Technology, Changchun, China

**Keywords:** academic perfectionism, Chinese adolescents, cross-cultural adaptation, differential item functioning, measurement invariance, perfectionistic concerns, psychometric refinement, SPS-4-CV

## Abstract

**Background:**

Academic perfectionistic concerns are distinct from perfectionistic strivings, yet existing brief study-related measures often conflate these facets and lack rigorous evaluation of measurement fairness. Using the Study-related Perfectionism Scale (SPS) as a starting point, we psychometrically refined and evaluated a concerns-focused four-item Chinese version (SPS-4-CV) among Chinese adolescents.

**Methods:**

A total of 1,126 junior middle school students were recruited using a three-independent-sample design. Sample 1 (*N* = 331, online) was used for item screening; Sample 2 (*N* = 333, paper-and-pencil) for exploratory factor analysis (EFA); and Sample 3 (*N* = 461, holdout) for confirmatory factor analysis (CFA) and fairness analyses. Polychoric EFA and WLSMV CFA were used to analyze ordinal data. Measurement invariance and Differential Item Functioning (DIF) were tested across gender and grade. A subset of Sample 3 (*N* = 133) completed a 7-week retest to assess test-retest reliability and longitudinal measurement invariance.

**Results:**

In Sample 3, ordinal CFA supported a robust unidimensional structure with excellent fit [χ^2^(2) = 2.07, *p* = 0.355; *CFI* = 0.999, *TLI* = 0.999, *RMSEA* = 0.009, *SRMR* = 0.017]. Standardized factor loadings ranged from 0.60 to 0.73. Scalar measurement invariance (equal loadings and thresholds) was supported across gender and grade. DIF analyses revealed no substantial bias, with negligible effect sizes (max Δ*NagelkerkeR*^2^ = 0.015). Internal consistency was acceptable (ordinal α = 0.754, ω_*total*_ = 0.755). Test-retest reliability was moderate (*ICC* = 0.560). Longitudinal analyses supported configural and metric invariance, as well as partial scalar invariance (partial threshold stability) over 7 weeks.

**Conclusion:**

The concerns-focused four-item Chinese version of the SPS (SPS-4-CV) is a psychometrically sound measure supported by rigorous evidence of internal structure, fairness (DIF), and longitudinal stability. It is suitable for research and group comparisons in educational settings and may serve as a brief indicator of potential academic perfectionistic concerns. While useful for screening, it is not intended for clinical diagnosis without further external validation.

## 1 Introduction

Academic perfectionism is increasingly studied in school settings because it can reflect both striving for excellence and concerns about mistakes, evaluation, and failure (Grugan et al., 2021). Recent measurement-focused studies have also emphasized that perfectionism is assessed using a diverse set of self-report instruments that vary in coverage and in the strength of their psychometric evidence (Lo et al., 2020). A widely used distinction separates perfectionistic strivings from perfectionistic concerns, with concerns reflecting a more maladaptive pattern of self-evaluation and threat sensitivity (Stoeber and Otto, 2006). This distinction is grounded in multidimensional models of perfectionism, such as those described by Frost et al. (1990) and Hewitt and Flett (1991). Meta-analytic evidence suggests that perfectionistic concerns show more consistent links with distress-related outcomes such as burnout, whereas associations for perfectionistic strivings are more variable across outcomes and contexts (Hill and Curran, 2016; Madigan, 2019; Curran and Hill, 2019). For research on student risk in educational contexts, a clear assessment of the concerns facet is therefore especially important.

Brief measures are attractive for school-based research and screening, yet they can be psychometrically fragile. A short scale covers less content and leaves little room to correct for an item that does not align with the intended construct. This increases the risk of construct underrepresentation and construct-irrelevant variance (Haynes et al., 1995). The risk is amplified when a brief instrument blends strivings and concerns at the item level, because the total score becomes difficult to interpret for research and group comparisons. When a measure is used across cultures or demographic groups, item-level evidence and measurement invariance tests are needed to support the claim that the same construct is assessed and that observed differences reflect substantive variation rather than measurement artifacts (Meredith, 1993). Applied guidance has further clarified recommended practices for invariance testing and reporting (Vandenberg and Lance, 2000; Millsap, 2011; Putnick and Bornstein, 2016). Simulation studies have shown that common fit indices can be sensitive to specific forms of lack of invariance (Chen, 2007).

These issues are particularly salient in Chinese educational settings. Despite recent policy reforms aimed at reducing academic burden (i.e., the “Double Reduction” policy), empirical evidence indicates that academic stress remains a primary correlate of depressive and anxiety symptoms among Chinese adolescents (Wang et al., 2024). Within this high-pressure context, achievement goals are often shaped by social-oriented motives and intense parental expectations (Cheung and Pomerantz, 2012; Li, 2002; Tao and Hong, 2014; Yang et al., 2025). Recent studies highlight that parent–child discrepancies in educational expectations are significant stressors that directly contribute to academic burnout and psychological distress (Fan and Hui, 2025; Liu et al., 2024). A recent study with Chinese adolescents also links perfectionism to school adjustment and highlights distinct roles for the self-oriented and socially prescribed dimensions (Jiang and Konorova, 2023; Luo et al., 2016). Consequently, striving for excellence may be experienced partly as an obligation to avoid disappointing significant others rather than as a purely self-chosen goal. We therefore expected that items framed as competitive excellence, such as aiming to be the best student or outperforming classmates, could function less as indicators of striving and more as indicators of evaluative concern, thereby blurring the distinction between facets and weakening the coherence of a brief scale. This concern directly motivated the item-level scrutiny in the present study: rather than assuming that all Study-related Perfectionism Scale (SPS) items would function equivalently in a Chinese adolescent sample, we treated the construct alignment of each candidate item—whether it primarily captures evaluative concern or competitive striving—as an empirical question to be resolved during screening.

We used the Study-related Perfectionism Scale (SPS), developed by Loscalzo et al. (2024), as a starting point because it was designed for school and learning contexts and is intentionally brief. Widely used multidimensional measures of perfectionism were developed as general trait instruments and are typically longer. This can limit the feasibility of large-scale school surveys and does not guarantee that item meanings will map cleanly onto a specific academic context. The SPS offers a concise, context-specific option, but its brevity makes it critical to evaluate whether each item functions as intended in a new cultural and educational setting. Building on evidence that perfectionistic concerns are more consistently linked to maladjustment, our goal was to derive a brief concerns-focused version rather than retain a mixed strivings-and-concerns score. In this study, we refined the SPS item set to develop a brief, concerns-focused Chinese version, aiming to maximize construct clarity. We refer to this refined Chinese version as the concerns-focused four-item Chinese version of the SPS (SPS-4-CV) (Study-related Perfectionism Scale, 4-item Chinese Version; concerns items only), which is derived from, and remains conceptually grounded in, the original SPS rather than constituting an independent instrument.

This study addressed the evidence gap about whether a brief, study-specific perfectionism measure yields a concern score with stable meaning among Chinese adolescents. We used a multi-sample design in which item screening, exploratory analysis, and confirmatory testing were conducted in separately collected student samples. We evaluated internal structure using EFA and CFA. Crucially, we tested scalar measurement invariance across gender and grade, and we conducted differential item functioning (DIF) analyses to ensure fairness. Furthermore, we examined longitudinal measurement invariance and test-retest reliability over a 7-week interval. Given that establishing a rigorous psychometric baseline is a prerequisite for clinical application, we intentionally focused our aims on construct refinement, internal structure, fairness evidence, and longitudinal stability, rather than on external clinical correlates at this stage. The SPS-4-CV is intended for research measurement and group comparisons in educational settings. It may also serve as a brief flag for elevated academic perfectionistic concerns that may develop over time regarding academic achievement and efficacy (Damian et al., 2017). However, it is not intended for clinical diagnosis, and we do not propose a hard cutoff.

## 2 Methods

### 2.1 Participants and procedure

Participants were Chinese junior middle school students in Grades 7–9 recruited from three schools in Inner Mongolia and Hubei Provinces. A convenience sampling approach was used. Schools were selected based on accessibility and willingness to cooperate with the research team. Within participating schools, Grade 7–9 students in the participating classes were invited to take part. Inclusion criteria were: (a) current enrollment in Grades 7–9 at a participating school and (b) provision of both written guardian consent and student assent. Students who did not provide both forms of consent/assent or were unavailable during the relevant administration window were not included. No additional screening criteria were applied. The study was conducted following institutional ethics approval and school permission. Written informed consent was obtained in accordance with local requirements for research involving minors, including the guardian's written consent and the student's assent.

A total of 1,126 students participated. To ensure a robust three-independent-samples design, we conducted three cross-sectional surveys, each collected separately. The three surveys did not overlap, and no student participated in more than one survey.

Sample 1 (*N* = 331) was collected via online administration and was used for item screening and initial construct refinement. Sample 2 (*N* = 333) was collected via paper-and-pencil administration and was used for exploratory factor analysis (EFA). Sample 3 (*N* = 462) was collected via survey and served as the independent holdout validation sample for confirmatory factor analysis (CFA), measurement invariance, and fairness analyses. Within this sample, 461 students provided complete responses on the SPS-4-CV items. Gender was available for *n* = 452 (222 male, 230 female). Grade distribution was Grade 7 (*n* = 163), Grade 8 (*n* = 133), and Grade 9 (*n* = 165). Sample 3 was strictly blinded from item selection and model development decisions.

Furthermore, a subset of Sample 3 (*n* = 201) completed additional perfectionism measures, including APS-R (Yang et al., 2007; Slaney et al., 2001) and FMPS subscales (Zi and Zhou, 2006; Frost et al., 1990), for within-domain association analyses. Furthermore, a specific subsample of Sample 3 (*n* = 133) completed the SPS-4-CV twice over a 7-week interval to evaluate test–retest reliability and longitudinal measurement invariance.

Surveys were administered in classroom groups during school hours. Responses were anonymous, and no incentives were provided. Since school and classroom identifiers were not retained in the analytic datasets, standard errors were not adjusted for clustering. Study samples and analytic roles are summarized in Table 1. The study involved human participants and was approved by the Ethics Committee of Minnan Normal University (Ref: 2025-06-09).

**Table 1:** Study samples and analytic roles.

Dataset	Analytic role	*n*	Notes
Sample 1	Item screening and construct refinement	331	Online administration
Sample 2	Polychoric EFA with common-factor extraction using MINRES	333	Paper and pencil administration
Sample 3	Ordinal CFA, invariance, and DIF	461	Held out from item selection. 462 were collected. Gender was available for 452.
Survey Subsample	Within-domain associations with additional perfectionism measures	201	Subset of Sample 3 with complete data on SPS-4-CV and correlates
Test–retest Subsample	ICC and longitudinal invariance across two waves	133	Subset of Sample 3. The retest interval was 7 weeks.

Samples were analyzed separately rather than pooled. We evaluated administration-mode effects as a sensitivity check using an ordinal multi-group CFA of the final SPS-4-CV. Results are reported in Supplementary Table 2. For each analysis, *n* denotes the number of participants with the required variables available.

### 2.2 Measure and translation procedure

The Study-related Perfectionism Scale (SPS; Loscalzo et al., 2024) is a brief 5-item measure designed to assess study-related perfectionism in school and learning contexts. With permission from the original authors, the full 5-item SPS item set was selected as the initial item pool. Items were adapted using a standard translation and back-translation process. Two bilingual psychology trainees conducted independent forward translations. Discrepancies were reconciled, and the Chinese version was back-translated and reviewed by the original author until conceptual equivalence was confirmed. Following the item screening process described in Section 2.3, four items were retained as the final adapted version, referred to hereafter as the SPS-4-CV (concerns items only).

Participants completed the Chinese version. Responses were recorded on a 5-point Likert-type scale ranging from 1 (strongly disagree) to 5 (strongly agree). For analysis, items were modeled as ordered categorical indicators.

For descriptive reporting and correlational analyses, we computed SPS-4-CV total scores by summing the responses to the four retained items when all four items were present. Higher scores indicate greater academic perfectionistic concerns. Table 2 presents the final SPS-4-CV items in Chinese together with back-translated English for reporting purposes.

**Table 2:** Final SPS-4-CV items in Chinese with back-translated English, response format, and scoring.

Item	Code	CFA loading	Back-translated English	Chinese wording
1	A1	0.648	I find it very difficult to accept even the slightest error on an exam.	考试中,哪怕是极小的错误也让 我非常难以接受。
2	A2	0.659	Being unable to answer a question makes me feel very ashamed.	回答不出问题,让我觉得很丢 人。
3	A3	0.734	If a classmate gets a higher score than me, I feel a sense of defeat.	如果有同学比我分数高,我会产 生失败感。
4	A5	0.598	Even if I get only one question wrong on an exam, I feel like I have completely failed at studying.	即使在考试中只答错一个题目,我也会觉得自己在学习上彻底失 败了。

Response options were 1, strongly disagree, 2, disagree, 3, neutral, 4, agree, and 5, strongly agree. Participants completed only the Chinese version. The English wording shown is a back-translation of the administered Chinese items, provided for reporting purposes; it is conceptually aligned with the item translations reported in Loscalzo et al. (2024) rather than the original SPS wording. SPS-4-CV total scores were computed by summing the four item responses when all four items were present. Possible scores range from 4 to 20. Higher scores indicate greater academic perfectionistic concerns. *CFA loading* is the standardized loading from the one-factor ordinal CFA in Sample 3.

### 2.3 Analytic strategy

All analyses were conducted in R version 4.5.1 (R Foundation for Statistical Computing, Vienna, Austria) using the psych package (William Revelle, Northwestern University, Evanston, IL, USA) (Revelle, 2025), lavaan package (Yves Rosseel, Ghent University, Ghent, Belgium) (Rosseel, 2012), semTools package (Terrence D. Jorgensen, University of Amsterdam, Amsterdam, The Netherlands, and contributors), and MASS package (Brian Ripley, Bill Venables, and contributors, CRAN/R Project), except longitudinal invariance, which was conducted in Mplus version 8.6 (Muthen and Muthen, 2017) to facilitate strict convergence checks for longitudinal ordinal data.

Item screening (Sample 1): We examined response distributions, corrected item–total correlations, and internal consistency changes if items were deleted. We also conducted extreme-groups comparisons (top vs. bottom 27%) to check item discrimination. Items were flagged for removal if they showed poor discrimination (corrected item–total correlation below 0.30), if internal consistency improved upon removal, or if item content was judged to be inconsistent with the target concerns construct based on conceptual review by the research team.

EFA (Sample 2): We computed polychoric correlations and used parallel analysis (Horn, 1965) to determine the number of factors. A common-factor EFA was then fitted using minimum residual (MINRES) extraction.

Ordinal CFA (Sample 3): We fitted a one-factor ordinal CFA with the WLSMV estimator and theta parameterization (Flora and Curran, 2004), appropriate for ordinal data. Model fit was evaluated using CFI, TLI, RMSEA, and SRMR, following commonly cited fit-index guidelines (Hu and Bentler, 1999). Given the brevity of the scale, we emphasized parameter estimates (loadings and thresholds) and residual patterns rather than global fit indices alone.

Fairness evidence: invariance and DIF (Sample 3): We tested ordinal measurement invariance across gender and grade using multi-group CFA. We evaluated configural, metric (loading), and scalar (threshold) invariance. Decisions relied primarily on changes in fit indices: a decrease in CFI (Δ*CFI*) of ≤ 0.010 and a change in RMSEA (Δ*RMSEA*) of ≤ 0.015 were considered evidence of invariance (Chen, 2007).

Differential item functioning (DIF) was evaluated using ordinal logistic regression models. For each item, the matching variable was the sum score of the remaining items to prevent part–whole contamination. We evaluated uniform and non-uniform DIF by assessing changes in Nagelkerke *R*^2^. Following Jodoin and Gierl (2001), effect sizes (Δ*R*^2^) below 0.035 were considered negligible.

Reliability and precision: Internal consistency was estimated using ordinal alpha and Omega Total (ω_*t*_; Dunn et al., 2014) based on the polychoric correlation matrix. Test–retest reliability was indexed by the Intraclass Correlation Coefficient [ICC [3,1]; two-way mixed effects]. A test information curve was derived from a graded response model to visualize precision across the latent trait.

Longitudinal invariance (retest subsample): To distinguish score stability from the stability of the measurement mechanism, we evaluated longitudinal invariance between Time 1 and Time 2. We tested configural, loading-invariant, and threshold-invariant models using the DIFFTEST procedure in Mplus with WLSMV estimation for nested model comparisons. When full threshold invariance proved too restrictive, we examined partial invariance.

Coding and missing data: In the raw data files, missing values were coded as 9 and were recoded as missing before analysis. Gender was coded as 1 for male and 2 for female. Grade was coded as 1 for Grade 7, 2 for Grade 8, and 3 for Grade 9. CFA, invariance, and DIF models used complete SPS-4-CV item responses. Group-based models also required non-missing group membership.

## 3 Results

We implemented a three-sample internal replication chain to separate item screening, exploratory analysis, and confirmatory testing. Sample 1 was used for item-level screening and construct refinement; Sample 2 for polychoric EFA; and Sample 3 for confirmatory testing and fairness analyses. Importantly, the final item set was locked after Sample 1, and Sample 3 was not used to make item selection decisions.

### 3.1 Item-level evidence and construct refinement

In Sample 1, which included *n* = 331 students, we screened the five candidate items, A1 through A5, using corrected item–total correlations and internal consistency diagnostics. We complemented these indices with an ordinal sensitivity check based on polychoric correlations and an extreme-groups discrimination test.

Across these indicators, SPS Item 4 (A4) showed the weakest evidence of discrimination. The corrected item–total correlation, *r*_*drop*_, was 0.291, whereas the other items ranged from 0.595 to 0.680. Although 0.291 was close to the common 0.30 screening benchmark, it was substantially lower than the other items. The same pattern emerged in the polychoric sensitivity check. The polychoric item–rest correlation for A4 was 0.276, and ordinal α for the five-item set increased to 0.864 when A4 was removed, as shown in Supplementary Table 14. Under numeric scoring, Cronbach's α also increased to 0.823 when A4 was removed.

For completeness, we also report the same screening patterns for the full five-item set in Samples 2 and 3. These analyses were conducted after the item set was finalized and are reported to document cross-sample consistency rather than to motivate further item selection. In Sample 2, A4 again had the smallest corrected item–total correlation, with *r*_*drop*_ equal to 0.128. In Sample 3, A4 remained comparatively weak, with *r*_*drop*_ equal to 0.369. In one-factor CFAs that treated responses as continuous, A4 also had the smallest standardized loading in both samples. In Sample 2, λ_*std*_ for A4 was 0.150. In Sample 3, λ_*std*_ for A4 was 0.457. Full results are shown in Supplementary Table 4.

Based on this combination of relative statistical weakness and content misalignment with a construct focused on concerns, we removed A4. We retained four items focused on concerns—A1, A2, A3, and A5—yielding the adapted Chinese version, hereafter referred to as the SPS-4-CV. Content review suggested that A4, “I want to be the best student in the class or a subject,” primarily reflected competitive striving rather than concern-related content. We did not retain A4 to index a separate striving dimension because a single indicator is insufficient to identify a latent factor.

As a complementary discrimination check in Sample 1, extreme-groups comparisons between lower and upper total-score groups showed large differences for all items. These groups were defined using cut scores near the 27th and 73rd percentiles, and ties increased the group sizes to *n*_*low*_ = 127 and *n*_*high*_ = 106, corresponding to 38.4% and 32.0% of the sample. The *t* values ranged from 7.82 to 22.65, and all *p* values were less than 0.001. This corresponded to large effect sizes, with *d* ranging from approximately 1.03 to 2.98. The removed item showed the weakest separation.

Item screening results are summarized in Table 3.

**Table 3:** Item-level screening results in Sample 1 (*n* = 331).

Item	*M*	*SD*	*r*_*drop*_	α if deleted	*t*	*p*
A1	2.662	1.070	0.611	0.727	17.761	< 0.001
A2	2.695	1.101	0.649	0.713	18.865	< 0.001
A3	2.622	1.114	0.680	0.702	22.649	< 0.001
A4	3.713	0.981	0.291	0.823	7.818	< 0.001
A5	2.085	0.937	0.595	0.736	12.129	< 0.001

Items used five response options. *r*_*drop*_ is the corrected item–total correlation, computed by scoring responses as numeric. “α if deleted” is Cronbach's α for the five-item set after removing the focal item, reported as a screening index. As an ordinal sensitivity check, the polychoric item–rest correlation for A4 was 0.276, and ordinal α if deleted was 0.864. *t* and *p*-values are from an extreme-groups test that compared lower and upper groups defined near the 27th and 73rd percentiles of the total-score distribution. Because of ties, the lower group included 127 students (38.4% of the sample), and the upper group included 106 students (32.0% of the sample). Item A4 was removed in deriving the four-item adapted Chinese version (SPS-4-CV).

### 3.2 Polychoric EFA and ordinal CFA

In Sample 2, which included *n* = 333 students, polychoric parallel analysis supported a single factor. The first two eigenvalues of the polychoric correlation matrix were 2.309 and 0.654, as shown in Supplementary Table 5. One-factor MINRES EFA yielded clear loadings for all retained items. These loadings ranged from 0.616 to 0.686, with acceptable communalities. Residuals were small, with an RMSR of 0.030.

Sample 3 included *n* = 461 students with complete SPS-4-CV responses. In this sample, ordinal CFA supported a unidimensional SPS-4-CV factor. Because a four-item one-factor model has only 2 degrees of freedom, near-perfect global fit indices are expected. We therefore interpreted fit alongside loadings and complementary diagnostic evidence.

Global fit indices suggested a close fit. The chi-square test was not significant, with χ^2^ equal to 2.07 and 2 degrees of freedom, and *p* equal to 0.355. The CFI and TLI were both 0.999. The RMSEA was 0.009, and the SRMR was 0.017. Standardized loadings ranged from 0.598 to 0.734, which corresponded to item *R*^2^ values of approximately 0.36 to 0.54, as shown in Supplementary Table 7. Item thresholds and standard errors are reported in Table 4. As a sensitivity check, we also fit a CFA that treated the items as continuous. Residual correlations were small, and the largest absolute residual correlation was below 0.04.

**Table 4:** SPS-4-CV ordinal CFA thresholds in Sample 3 (WLSMV, theta).

Item	*t*_1_	SE	*t*_2_	SE	*t*_3_	SE	*t*_4_	SE
A1	–1.491	0.105	–0.321	0.078	0.835	0.085	2.033	0.131
A2	–1.757	0.115	–0.586	0.082	0.222	0.078	1.658	0.108
A3	–1.768	0.122	–0.245	0.086	0.667	0.092	2.307	0.144
A5	–0.728	0.078	0.768	0.081	1.788	0.111	2.340	0.145

Thresholds are reported on the underlying continuous response scale from the one-factor ordinal CFA in Sample 3. *t*_1_ through *t*_4_ correspond to the thresholds between the five ordered response categories.

EFA and CFA loadings are summarized in Table 5.

**Table 5:** Unidimensional structure of the SPS-4-CV.

	Sample 2 (EFA)		Sample 3 (CFA)
Item	Loading	*h* ^2^	Standardized loading
A1	0.616	0.380	0.648
A2	0.686	0.471	0.659
A3	0.674	0.455	0.734
A5	0.665	0.442	0.598

EFA used polychoric correlations with MINRES extraction (Sample 2; *n* = 333). CFA used WLSMV (theta) (Sample 3; *n* = 461 complete). Item wordings are shown in Table 2. CFA fit was χ^2^(2) = 2.07, *p* = 0.355, with CFI = 0.999, TLI = 0.999, RMSEA = 0.009, and SRMR = 0.017. With four indicators and a one-factor model, the CFA has very low degrees of freedom (*df* = 2), so near-perfect global fit indices can occur. Interpretation, therefore, emphasizes parameter estimates and evidence of complementary validity.

### 3.3 Administration-mode sensitivity check

To evaluate administration-mode sensitivity, we fitted an ordinal two-group CFA of the final SPS-4-CV. Because the administration mode was confounded with the sampling context, this comparison serves as a sensitivity analysis rather than a causal test. Sample 1 was administered online, whereas Sample 2 was administered using paper and pencil.

The configural model fits well. Loading invariance was supported. Threshold invariance was not supported, so a partial threshold-invariance model was required. In this partial model, we freed the first two thresholds of Item A5 and obtained a close-fitting solution. Descriptively, the lowest category of A5 was endorsed slightly more often during paper administration (32.1%) than during online administration (28.4%). Full model-fit details are shown in Supplementary Table 2, and descriptive response distributions are shown in Supplementary Table 3. Because this is a sensitivity analysis, nested-model chi-square difference testing under WLSMV depends on the scaling approach. Hence, conclusions emphasized changes in approximate fit indices rather than chi-square difference tests. This pattern should not be interpreted as causal evidence of administration-mode effects.

### 3.4 Fairness evidence: ordinal invariance and DIF

In Sample 3, ordinal multi-group CFA supported invariance across gender and grade, as indicated by changes in approximate fit indices. These conclusions were further corroborated by DIF analyses. The gender models used data from 452 students, including 222 boys and 230 girls. The grade models used 461 students, including 163 seventh graders, 133 eighth graders, and 165 ninth graders. Across both groupings, changes in CFI were very small and did not exceed 0.002, and SRMR changes were modest, as shown in Table 6. For grade, RMSEA increased by 0.032 at the loading-equality step. However, RMSEA can be unstable in models with few degrees of freedom, particularly when the baseline RMSEA is near zero (Kenny et al., 2015; MacCallum et al., 1996). We therefore emphasized changes in CFI, SRMR, and the overall DIF pattern when evaluating invariance. These CFI values were also well below the 0.01 guideline commonly used in invariance evaluations (Chen, 2007).

**Table 6:** Fairness evidence from ordinal multi-group CFA and DIF analyses.

	Model fit						Invariance tests			
Model	χ^2^	df	CFI	TLI	RMSEA	SRMR	Δdf	ΔCFI	ΔRMSEA	ΔSRMR
Gender										
Configural	3.668	4	1.000	1.000	0.000	0.023				
Loading invariance	7.113	7	1.000	1.000	0.009	0.032	3	0.000	0.009	0.009
Threshold invariance	17.323	18	1.000	1.000	0.000	0.028	11	0.000	–0.009	–0.004
Grade										
Configural	4.227	6	1.000	1.000	0.000	0.024				
Loading invariance	13.821	12	0.998	0.997	0.032	0.041	6	–0.002	0.032	0.017
Threshold invariance	29.303	34	1.000	1.000	0.000	0.036	22	0.002	–0.032	-0.006

CFAs used ordinal WLSMV estimation. We evaluated invariance using changes in CFI and SRMR, and we interpreted chi-square differences cautiously. Fit indices and changes are shown to 3 decimal places. DIF was tested with ordinal logistic regression. The maximum change in Nagelkerke *R*^2^ was 0.015, which is below the 0.035 cutoff commonly used to classify DIF as negligible (Jodoin and Gierl, 2001). For gender, uniform $\Delta R_{max}^{2}$ was 0.007 for A5 and non-uniform $\Delta R_{max}^{2}$ was 0.009 for A1. For grade, uniform $\Delta R_{max}^{2}$ was 0.014 for A2 and non-uniform $\Delta R_{max}^{2}$ was 0.009 for A5.

DIF analyses also supported fairness. Effect sizes were small for both uniform and non-uniform DIF across gender and grade. The maximum change in Nagelkerke *R*^2^ was 0.015, which was below the 0.035 threshold used for negligible DIF. Taken together, the invariance and DIF results suggest that observed group comparisons are unlikely to be meaningfully distorted by item-level bias. Full item-level results are provided in Supplementary Table 8.

### 3.5 Within-domain associations

The survey subsample included *n* = 201 students who completed additional perfectionism measures. Table 7 summarizes key Pearson correlations between SPS-4-CV scores and concern-related perfectionism subscales, with Fisher-*z* confidence intervals. The full set of correlations is reported in Supplementary Table 10. Overall, the pattern was consistent with a concerns-focused interpretation. Associations with order and organization indices were smaller, as shown in Supplementary Table 10.

**Table 7:** Key within-domain correlations between SPS-4-CV total score and other perfectionism subscales (survey subsample).

Target scale/subscale	*r*	95% CI	*n*
APS-R discrepancy	0.562	0.459 to 0.650	201
CFMPS concern over mistakes	0.373	0.248 to 0.487	201
CFMPS doubts about actions	0.261	0.127 to 0.386	201

Correlations are Pearson correlations. Confidence intervals are Fisher-*z* intervals. Full correlation results, including additional subscales and false discovery rate adjusted *p* values, are reported in Supplementary Table 9.

### 3.6 Reliability and precision

In Sample 3, SPS-4-CV total scores averaged 10.84 with a standard deviation of 3.17, spanning the full possible range from 4 to 20. Floor and ceiling rates were low, as shown in Table 8.

**Table 8:** Descriptive statistics for SPS-4-CV total scores in Sample 3.

Group	*n*	*M*	*SD*	Range	Floor (%)	Ceiling (%)
Overall	461	10.84	3.17	4–20	4.3	0.9
Male	222	10.37	3.42	4–20	7.2	1.4
Female	230	11.33	2.86	4–20	1.7	0.4
Grade 7	163	10.95	3.29	4–20	4.3	1.2
Grade 8	133	11.07	3.15	4–20	3.8	0.8
Grade 9	165	10.55	3.07	4–20	4.8	0.6

SPS-4-CV total scores range from 4 to 20. Floor and ceiling percentages are the proportions of participants scoring 4 and 20, respectively. Gender summaries are based on participants with non-missing gender data.

**Figure 1:**
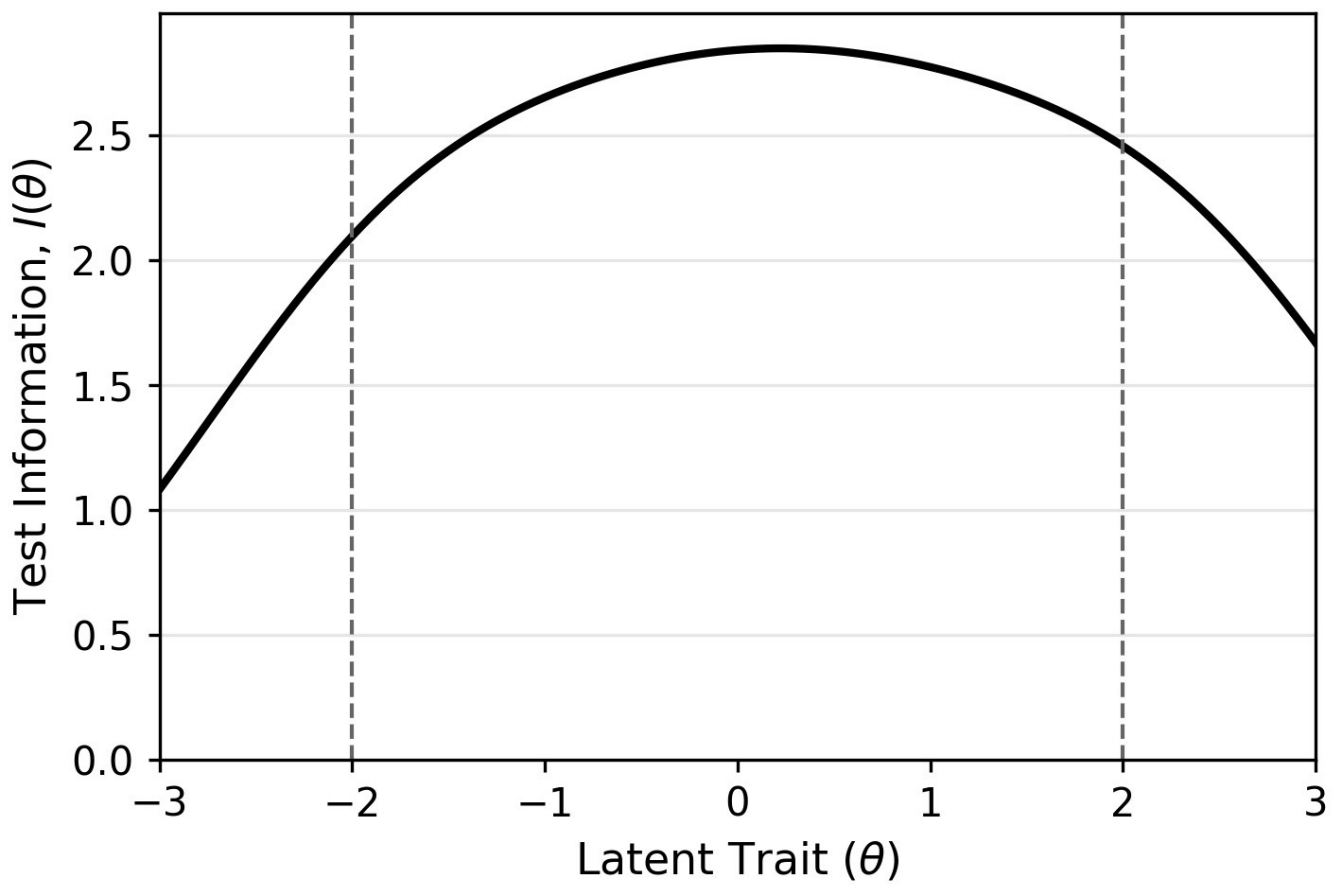
Test information function for the 4-item SPS-4-CV based on Sample 3. Higher values indicate greater precision at that trait level. Precision was highest in the central range of the trait and declined toward the extremes. Dashed vertical lines mark θ = −2 and θ = 2.

Internal consistency was acceptable. Ordinal α based on polychoric correlations was 0.754, and omega total, ω_*t*_, was 0.755. The test information curve (Figure 1) suggested that the SPS-4-CV is most precise around the typical range of the latent trait. Peak information occurred at θ≈0.20. Information remained adequate within the range of θ≈−2 to θ≈2, covering the majority of the latent trait distribution. Precision was lower at the extremes of the trait distribution, for example, when θ was below approximately −2 or above approximately 2.

### 3.7 Test-retest reliability and longitudinal invariance

The test-retest subsample included *n* = 133 students from Sample 3 who completed the SPS-4-CV twice over 7 weeks. The SPS-4-CV total-score ICC(3,1) was 0.560. The 95% confidence interval ranged from 0.431 to 0.666.

We evaluated longitudinal invariance in Mplus using WLSMV with theta parameterization and DIFFTEST (Satorra, 2000). The configural model fit adequately. The chi-square test was χ^2^(16) = 26.07 with *p* = 0.053. The CFI was 0.990, and the TLI was 0.982. The RMSEA was 0.069, and the SRMR was 0.032. Loading invariance was supported relative to the configural model. The DIFFTEST chi-square difference test was χ^2^(3) = 4.08 with *p* = 0.253. Full threshold invariance was not supported. The DIFFTEST chi-square difference test was χ^2^(16) = 37.15 with *p* = 0.002, indicating selective threshold shifts between waves.

A partial threshold-invariance model constrained thresholds to equality for two empirically stable items, A2 and A5. This partial model did not significantly worsen fit relative to the loading-invariant model. The DIFFTEST chi-square difference test was χ^2^(8) = 11.53 with *p* = 0.173, supporting longitudinal comparability anchored by invariant thresholds. In this partial model, the latent test–retest correlation was 0.606 in the STDYX metric. Threshold drift was concentrated in the non-anchored items, A1 and A3, as shown in Supplementary Table 12.

Longitudinal measurement invariance model-fit results are summarized in Table 9.

**Table 9:** Longitudinal measurement invariance results (T1 vs. T2) from Mplus (WLSMV, theta; DIFFTEST) in the 7-week retest subsample (*n* = 133).

	Model fit							DIFFTEST		Change	
Model	χ^2^	df	*p*	CFI	TLI	RMSEA	SRMR	χ^2^	df	ΔCFI	ΔRMSEA
Configural	26.070	16	0.053	0.990	0.982	0.069	0.032				
Loadings equal	29.331	19	0.061	0.989	0.984	0.064	0.036	4.084	3	–0.001	–0.005
Thresholds equal (full)	64.539	35	0.002	0.970	0.976	0.080	0.042	37.151	16	–0.019	0.016
Thresholds equal											
(partial; A2 and A5)	39.974	27	0.052	0.987	0.986	0.060	0.038	11.530	8	–0.002	–0.004

DIFFTEST comparisons are relative to the immediately less constrained model, except for the partial threshold-invariance model, which is compared against the loading-invariance model. ΔCFI and ΔRMSEA are shown as descriptive invariance diagnostics (Chen, 2007).

## 4 Discussion

This study adapted the Study-related Perfectionism Scale (SPS; Loscalzo et al., 2024) through translation/back-translation, combined with item- and construct-level psychometric evaluation, rather than relying solely on a direct translation. The present study is best characterized as a cultural adaptation and psychometric refinement of the SPS rather than *de novo* scale development. The primary contribution lies in deriving and validating an SPS-4-CV, supported by rigorous evidence for internal structure, psychometric fairness, and longitudinal comparability. Evidence at the item level indicated construct heterogeneity: one item that plausibly reflected perfectionistic strivings did not align psychometrically with the remaining concern-focused items among Chinese adolescents. Removing this item yielded a concise four-item concerns-focused Chinese version (SPS-4-CV) that focuses specifically on maladaptive concerns and supports fair research measurement and group comparisons.

Across EFA and CFA that treated responses as ordered categories, the SPS-4-CV showed a stable unidimensional structure with strong loadings. Crucially, fairness evidence was supported in two complementary ways. We found ordinal measurement invariance across gender and grade, including scalar invariance (i.e., invariant thresholds), and negligible differential item functioning (DIF) effect sizes. This suggests that the scale functions comparably across key demographic subgroups, a prerequisite for unbiased group comparisons. Within-domain associations with established perfectionism subscales followed a concerns-consistent pattern, with stronger correlations for discrepancy and concern over mistakes than for organization and order. Thus, the adaptation and refinement of the SPS was not an end in itself, but the psychometric route through which we obtained a concerns-focused score whose interpretation could be evaluated. In the present Chinese junior-middle-school samples, the evidence supports a largely stable concerns-score interpretation, as indicated by a consistent one-factor structure in independent EFA and CFA samples, scalar invariance across gender and grade, negligible DIF, concerns-consistent within-domain associations, and partial longitudinal threshold invariance over 7 weeks.

A key implication of this refinement is interpretability. An SPS-4-CV that focuses strictly on concerns aligns more closely with the facet of perfectionism that is most consistently linked to maladjustment in academic settings (Stoeber and Otto, 2006; Hill and Curran, 2016; Madigan, 2019; Curran and Hill, 2019). The removed item (“I want to be the best student...”) likely reflected a culturally normative striving for excellence rather than maladaptive concern. In competitive educational contexts, such goals may be socially reinforced and less indicative of distress, which would help explain the item's weaker discrimination and mismatch with the concerns-focused core. By purifying the construct, the SPS-4-CV avoids mixing of facets that complicates the interpretation of brief measures (Haynes et al., 1995; Stoeber and Gaudreau, 2017). Our approach is consistent with the translation-plus-psychometric re-evaluation observed in the French-Canadian adaptation study by Vigneau and colleagues. For example, French et al. (2005) reported a French-Canadian adaptation of the Pain Catastrophizing Scale whose dimensional structure only partially reproduced the original, while Rousselle and Vigneau (2016) identified redundant items in the French BIS-15 and consequently favored a unidimensional model with correlated residuals. These examples illustrate that cross-language adaptation can legitimately include empirical item-level and structural refinement when construct representation shifts in the target population. Researchers should note that the SPS-4-CV assesses only the concerns facet of the original SPS; it should not be treated as interchangeable with the full 5-item SPS, which also includes a strivings item.

### 4.1 Interpreting longitudinal stability and change

The test–retest reliability (*ICC* = 0.560) over 7 weeks was moderate, but this should not be interpreted as psychometric instability. Stability indices based on observed scores conflate true change, transient state effects, and measurement error. Longitudinal invariance modeling provided deeper insight. The measurement mechanism remained stable in structure and loadings (metric invariance), whereas full threshold invariance was overly restrictive. Partial threshold invariance was supported when stable items (A2 and A5) were used as anchors.

This pattern is theoretically consistent with meaningful short-term variability in academic perfectionistic concerns. In school contexts, threshold shifts may reflect changes in academic demands over time, such as periods near major exams or after receiving performance feedback. Practically, this implies that raw total-score changes should be interpreted with caution. When change over time is of interest, longitudinal comparisons are better conducted using latent variable models that accommodate partial invariance, rather than relying on simple raw-score differences.

Conceptually, the SPS-4-CV is best interpreted as a brief index of academic evaluative concerns, including intolerance of mistakes, shame in performance situations, and negative self-evaluation, rather than a comprehensive assessment of the broad perfectionism construct. Its content is intentionally narrow and context-specific. This enhances the feasibility of large-scale school surveys but entails a trade-off in content breadth (Lo et al., 2020). For example, facets reflecting externally imposed pressure, such as parental expectations, are not explicitly covered, though they can be relevant in Chinese educational settings (Jiang and Konorova, 2023). Thus, the refined SPS-4-CV offers clearer signal clarity at the cost of a narrower bandwidth.

### 4.2 Limitations and future directions

Several limitations should be noted. First, this study emphasized construct refinement, internal structure, and evidence of fairness. We did not include external criteria such as clinical outcomes, teacher ratings, or objective achievement. Accordingly, conclusions regarding the scale's utility should be bound to research measurement and group comparisons within the studied context. Future studies must test predictive and incremental validity against external criteria before making screening claims. Specifically, we recommend examining associations with test anxiety, academic burnout, and objective academic performance to establish the scale's criterion-related utility.

Second, while we established invariance by gender and grade, the homogeneity of our sample limits the generalizability to other regions, socioeconomic statuses, and school types. Cross-cultural or cross-regional invariance testing remains a necessary step to confirm the scale's applicability in broader Chinese populations.

Additionally, because school and classroom identifiers were not retained in the analytic datasets to protect anonymity, we could not adjust standard errors for potential clustering effects (i.e., a nested data structure). Ignoring clustering can lead to underestimated standard errors and inflated Type I error rates in significance testing. To mitigate this risk, we prioritized fit indices (e.g., CFI, RMSEA) and effect sizes (e.g., Δ Nagelkerke *R*^2^ for DIF) over statistical significance tests when evaluating model fit and measurement invariance. Although parameter estimates (loadings and thresholds) generally remain consistent even without clustering adjustments, future studies should retain multilevel identifiers to allow for robust standard error estimation.

Third, exact chronological age was not collected; grade level was used as a proxy for developmental stage. In the standardized Chinese education system, grade is strongly correlated with age, but future studies should collect age data directly to enable more granular developmental analyses.

Fourth, the subsamples differed in administration mode (online vs. paper-and-pencil). Although our sensitivity check supported comparable factor loadings, it indicated modest differences in the thresholds for the lowest response category of Item A5. This cautions against treating observed mean differences as strictly comparable across administration modes without testing for scalar invariance.

Finally, longitudinal results supported only partial threshold invariance. Future studies should evaluate the sources of threshold shifts, including exam-related time effects, and replicate these findings in larger, multi-wave longitudinal samples.

In conclusion, the SPS-4-CV provides a psychometrically sound, concerns-focused index for Chinese adolescents, supported by rigorous evidence of internal structure, measurement fairness (DIF), and longitudinal stability. Its primary strengths are construct clarity and efficiency. While it shows promise as a research tool for group comparisons, future validation against clinical and educational outcomes is necessary to establish its utility for individual screening.

## Data Availability

The raw data supporting the conclusions of this article will be made available by the authors, without undue reservation.
