# Supplementary material for: Psychometric refinement of the study-related perfectionism scale in Chinese adolescents: fairness and longitudinal evidence for a four-item concerns-focused adaptation (SPS-4-CV)
Source: Front Psychol. 2026 Jul 3;17:1789348. doi: 10.3389/fpsyg.2026.1789348 (PMC13377225; doi:10.3389/fpsyg.2026.1789348)
Supplement: Supplementary file 1 [file Data_Sheet_1.pdf]

Supplementary Material

SUPPLEMENTARY DATA

Appendix A: Study-related Perfectionism Scale, 4-item Chinese Version (SPS-4-CV)

学业完美主义担忧问卷 **(SPS-4-CV)**

指导语：本问卷旨在评估你对学习的态度。请阅读下列陈述，并结合你在学校的情境回答。在作答时，请回想你通常是怎样准备考试的。所有答案没有对错之分，请尽可能诚实地回答所有问题。感谢你的参与。

作答方式：1= 非常不同意；2= 不同意；3= 一般；4= 同意；5= 非常同意。

条目：

1. A1: 考试中，哪怕是极小的错误也让我非常难以接受。
2. A2: 回答不出问题，让我觉得很丢人。
3. A3: 如果有同学比我分数高，我会产生失败感。
4. A5: 即使在考试中只答错一个题目，我也会觉得自己在学习上彻底失败了。

Appendix B: SPS-4-CV items (back-translated English for international readers)

Response options: 1=Strongly disagree; 2=Disagree; 3=Neutral; 4=Agree; 5=Strongly agree. The SPS-4-CV was administered in Chinese. The English wording below is a back-translation provided to support international reporting and reuse. The original SPS English item wording is available in Loscalzo et al., 2024 and is not reproduced here.

1. **A1:** In exams, even tiny mistakes are very hard for me to accept.
2. **A2:** Not being able to answer a question makes me feel ashamed.
3. **A3:** If a classmate scores higher than me, I feel like a failure.
4. **A5:** Even if I get only one question wrong on an exam, I feel I have completely failed in my studies.

Candidate item removed during screening (English translation)

1. **A4:** I want to be the best student in the class or a subject.

Notes on longitudinal invariance modeling In a 7-week test-retest subset of Sample 3 with n = 133, longitudinal invariance between Time 1 and Time 2 was evaluated in Mplus using WLSMV with theta parameterization and DIFFTEST. The partial threshold invariance model constrained thresholds for items **A2** and **A5**, while allowing the remaining item thresholds to vary.

SUPPLEMENTARY TABLES

Table S1. Comparison of items between the original Study-related Perfectionism Scale (SPS) and the adapted SPS-4-CV (concerns items only).

Original Item (SPS)	Loading[a]	Chinese Wording	Decision	Rationale
1. In exams, even tiny mistakes are very hard for me to accept.	.61	考试中，哪怕是极小的错误也让我非常难以接受。	**Retained** (Item A1)	High discrimination; reflects intolerance of mistakes.
2. Not being able to answer a question makes me feel ashamed.	.55	回答不出问题，让我觉得很丢人。	**Retained** (Item A2)	High discrimination; reflects shame/fear of negative evaluation.
3. If a classmate scores higher than me, I feel like a failure.	.69	如果有同学比我分数高，我会产生失败感。	**Retained** (Item A3)	High discrimination; reflects sensitivity to social comparison failure.
4. I want to be the best student in the class or a subject.	.63	我想成为班级/某课中最优秀的学生。	**Removed**	Low discrimination ($r_{drop} = .29$); conceptually reflects "Striving" (positive ambition) rather than "Maladaptive Concern" in the Chinese context.
5. Even if I get only one question wrong on an exam, I feel I have completely failed in my studies.	.74	即使在考试中只答错一个题目，我也会觉得自己在学习上彻底失败了。	**Retained** (Item A5)	High discrimination; reflects strict self-evaluation standards.

Note. [a] Original standardized factor loadings are from Loscalzo et al. (2024). The Chinese items were used in the initial screening (Sample 1). Item 4 was removed to purify the construct, resulting in the final 4-item SPS-4-CV.

Table S2. Administration-mode invariance results comparing online and paper and pencil administrations of the SPS-4-CV in Sample 1 and Sample 2. Models were estimated with WLSMV using theta parameterization in lavaan. For WLSMV, we report the scaled test statistic and scaled incremental and approximate fit indices. Difference tests use a scaled chi-square difference test following Satorra (2000).

Model	Scaled χ^2	df	CFI	TLI	RMSEA	SRMR	$\Delta\chi^2$	Δdf	p	Notes
Configural	8.371	4	0.998	0.993	0.057	0.016				
Loadings equal	7.919	7	1.000	0.999	0.020	0.018	1.147	3	0.766	vs. configural
Thresholds equal (full)	34.524	18	0.992	0.995	0.053	0.019	26.460	11	0.006	vs. loadings
Thresholds equal (partial)	18.145	16	0.999	0.999	0.020	0.019	10.737	9	0.294	vs. loadings; freed A5|t1 and A5|t2

Table S3. Item A5 response distributions and marginal thresholds by administration mode (Sample 1 online vs. Sample 2 paper and pencil).

	Online (Sample 1; $n = 331$)		Paper (Sample 2; $n = 333$)	
Category	n	%	n	%
1	94	28.4	107	32.1
2	147	44.4	139	41.7
3	64	19.3	53	15.9
4	20	6.0	21	6.3
5	6	1.8	13	3.9

Note. Marginal thresholds are descriptive probit cutpoints computed from cumulative response proportions (with a continuity correction); they summarize mode-associated shifts in category usage but do not adjust for latent-trait differences between samples. *Marginal thresholds (A5):* Online $t_1 = -0.575$, $t_2 = 0.603$, $t_3 = 1.405$, $t_4 = 2.061$. Paper $t_1 = -0.468$, $t_2 = 0.635$, $t_3 = 1.261$, $t_4 = 1.744$. The paper administration had slightly higher values for the lowest thresholds. The difference was 0.107 for t_1 and 0.032 for t_2.

Table S4. Cross-sample sensitivity evidence for the removed item A4 (SPS Item 4) based on the full five-item set.

Sample	n	α (5 items)	A4 r_{drop}	α if A4 deleted	A4 λ_{std}	λ_{std} min
Sample 1 (online)	331	0.785	0.291	0.823	0.315	yes
Sample 2 (paper)	333	0.644	0.128	0.706	0.150	yes
Sample 3 (held-out)	461	0.715	0.369	0.709	0.457	yes

Note. r_{drop} is the corrected item–total correlation (item–rest correlation) computed from raw responses. λ_{std} is the standardized loading for A4 from a one-factor five-indicator CFA treating responses as continuous (ML; semopy). "λ_{std} min" indicates whether A4 was the smallest standardized loading among the five items in that sample.

Table S5. Additional details for the Sample 2 polychoric EFA (four retained SPS-4-CV items).

	Polychoric correlation matrix eigenvalues			
Item set	λ_1	λ_2	λ_3	λ_4
A1, A2, A3, A5	2.309	0.654	0.541	0.497

Note. Parallel analysis suggested 1 factor. Residuals: RMSR (off-diagonal; implied by MINRES loadings) = 0.030.

Table S6. SPS-4-CV ordinal CFA thresholds (Sample 3; WLSMV, theta).

Item	Threshold	Estimate	SE	p
A1	t_1	-1.491	0.105	$< .001$
	t_2	-0.321	0.078	$< .001$
	t_3	0.835	0.085	$< .001$
	t_4	2.033	0.131	$< .001$
A2	t_1	-1.757	0.115	$< .001$
	t_2	-0.586	0.082	$< .001$
	t_3	0.222	0.078	0.005
	t_4	1.658	0.108	$< .001$
A3	t_1	-1.768	0.122	$< .001$
	t_2	-0.245	0.086	0.004
	t_3	0.667	0.092	$< .001$
	t_4	2.307	0.144	$< .001$
A5	t_1	-0.728	0.078	$< .001$
	t_2	0.768	0.081	$< .001$
	t_3	1.788	0.111	$< .001$
	t_4	2.340	0.145	$< .001$

Table S7. CFA diagnostic information for the SPS-4-CV in Sample 3.

Item	Standardized loading	Implied R^2
A1	0.648	0.420
A2	0.659	0.434
A3	0.734	0.539
A5	0.598	0.358

Note. Implied R^2 values are computed as squared standardized loadings from the ordinal CFA reported in the main manuscript. Residual indices are from a sensitivity CFA treating the four items as continuous and are reported as a descriptive check on local dependence. Continuous-treatment CFA residuals (Sample 3; ML; semopy): max $|r_{res}| = 0.037$; RMSR = 0.023.

Table S8. DIF results from ordinal logistic regression.

Grouping	Item	n	Uniform ΔR^2	p	Nonuniform ΔR^2	p
Gender	A1	452	0.0022	0.339	0.0091	0.050
	A2	452	0.0007	0.583	0.0032	0.248
	A3	452	0.0064	0.103	0.0001	0.855
	A5	452	0.0072	0.087	0.0039	0.209
Grade	A1	461	0.0002	0.785	0.0062	0.266
	A2	461	0.0145	0.012	0.0031	0.511
	A3	461	0.0016	0.404	0.0009	0.832
	A5	461	0.0091	0.052	0.0086	0.167

Table S9. Normal-ogive IRT parameters for the SPS-4-CV (Sample 3).

Item	a	b_1	b_2	b_3	b_4
A1	0.861	-1.499	-0.323	0.839	2.044
A2	0.863	-1.744	-0.582	0.220	1.646
A3	1.086	-1.772	-0.246	0.668	2.313
A5	0.742	-0.726	0.767	1.785	2.336

Table S10. Within-domain nomological correlations between SPS-4-CV total score and other perfectionism subscales (survey subsample; $n = 201$).

Target scale/subscale	r	95% CI (lower)	95% CI (upper)	FDR p
APS-R Standards (APSR_ST)	0.515	0.405	0.610	$< .001$
APS-R Order (APSR_OR)	0.329	0.199	0.447	$< .001$
APS-R Discrepancy (APSR_DI)	0.562	0.459	0.650	$< .001$
CFMPS Concern over Mistakes (CFMPS_CM)	0.373	0.248	0.487	$< .001$
CFMPS Organization (CFMPS_OR)	0.140	0.001	0.273	0.048
CFMPS Parental Expectations (CFMPS_PE)	0.227	0.092	0.355	0.001
CFMPS Personal Standards (CFMPS_PS)	0.313	0.182	0.433	$< .001$
CFMPS Doubts about Actions (CFMPS_DA)	0.261	0.127	0.386	$< .001$

Note. Confidence intervals are Fisher-z intervals. p values are adjusted using the Benjamini–Hochberg false discovery rate procedure. Planned comparisons of dependent correlations did not support a clear difference between SPS-4-CV correlations with APS-R Discrepancy and APS-R Standards, $t = 0.962$, $p = .337$, or between CFMPS Doubts about Actions and CFMPS Organization, $t = 1.548$, $p = .123$.

Table S11. Longitudinal invariance results (T1 vs. T2) from Mplus (WLSMV, theta; DIFFTEST).

	Model fit							DIFFTEST	
Model	χ^2	df	p	CFI	TLI	RMSEA	SRMR	χ^2	df
Configural	26.070	16	.053	.990	.982	.069	.032		
Loadings equal	29.331	19	.061	.989	.984	.064	.036	4.084	3
Thresholds equal (full)	64.539	35	.002	.970	.976	.080	.042	37.151	16
Thresholds equal (partial; A2 and A5)	39.974	27	.052	.987	.986	.060	.038	11.530	8

Table S12. Threshold drift between Time 1 and Time 2 in the partial longitudinal invariance model (Mplus WLSMV, theta; anchored A2 and A5).

Item	Threshold	T1 estimate	T2 estimate	T2–T1
A1	t_1	-0.962	-0.645	0.317
	t_2	0.201	0.508	0.307
	t_3	1.292	1.998	0.706
	t_4	2.658	3.310	0.652
A2	t_1	-1.086	-1.086	0.000
	t_2	-0.214	-0.214	0.000
	t_3	0.596	0.596	0.000
	t_4	1.812	1.812	0.000
A3	t_1	-1.345	-1.024	0.321
	t_2	0.071	0.419	0.348
	t_3	1.041	1.856	0.815
	t_4	3.145	5.070	1.925
A5	t_1	-0.120	-0.120	0.000
	t_2	1.184	1.184	0.000
	t_3	2.297	2.297	0.000
	t_4	2.650	2.650	0.000

Note. Thresholds are on the probit scale from the partial threshold-invariance model in which thresholds for A2 and A5 were constrained equal across waves. In this model, the latent test–retest correlation was STDYX $r = 0.606$.

Table S13. Test-retest reliability over a 7-week interval in a subsample of $n = 133$ using intraclass correlation coefficients, ICC.

Type	ICC	95% CI (lower)	95% CI (upper)
ICC(1,1)	0.543	0.411	0.652
ICC(2,1)	0.548	0.414	0.658
ICC(3,1)	0.560	0.431	0.666
ICC(1,k)	0.704	0.583	0.789
ICC(2,k)	0.708	0.585	0.794
ICC(3,k)	0.718	0.603	0.800

Table S14. Ordinal screening indices for the five-item pool in Sample 1 ($n = 331$).

Item	Polychoric item–rest correlation	Ordinal α if deleted
A1	0.672	0.750
A2	0.703	0.741
A3	0.745	0.727
A4	0.276	0.864
A5	0.632	0.763

Note. Polychoric correlations were estimated under a bivariate normal threshold model. Item–rest correlations were computed from the polychoric correlation matrix as the correlation between the focal item and the sum of the remaining items. Ordinal α values were computed from the same polychoric correlation matrix.
